# The First Session of the Research Society of the American Red Cross in France

**Published:** 1918-01

**Authors:** 


### The Medical Bulletin

N° 3 Paris January 1918

### RESEARCH SOCIETY REPORTS

### The First Session of the Research Society of the American Red Cross in France

December 11 and 12, 1917, at 6, rue Piccini, Paris.

The meeting was called to order by Major Lambert, Chief Surgeon of the American Red Cross in France. He spoke briefly of the aims of the Research Society as outlined in the Announcement prefacing the first number of the Medical Bulletin. He introduced Major Murphy, chief of the American Red Cross in Europe, who assured the Society of his keen interest and support. He believed the Society would be valuable in aiding the American physicians to prepare for coming events, and also would serve as a further means of contact between our Allies and ourselves, thus, perhaps, enabling us, in some measure, to repay the very great courtesy and generosity shown by the Allies towards American workers in all branches of war service.

Major Lambert then requested Major Blake, U. S. R., to take the chair during the scientific part of the session. Major Blake announced that the subject for the first meeting of the session was Gas Gangrene. Incidentally he called the attention of the meeting to the fact that a Research Committee has heen appointed, of which Dr. Kenneth Taylor, 6, rue Piccini, is the secretary. This Committee would be glad to receive suggestions concerning the plans for meetings of the Society, or concerning the research work to be carried on. He expressed the hope that the research workers of the allied nations would cooperate. He then introduced the first speaker, Colonel Cuthbert Wallace, R. A. M. C.

Colonel Wallace stated that he would present the clinical picture of gas gangrene, and that his colleagues, Captain Henry and Major McNee would deal with its bacteriology and microscopic anatomy.

The speaker then drew a picture of the disease as it occurred in untreated cases. It could be divided on a clinico-pathological basis into two types — the *group* type and the *segmental* type.

*The Group Type.* A patient arrived in hospital with a single entry shell wound of the thigh. It was dressed and the patient put to bed. In a few hours the patient felt uncomfortable and experienced some pain in the limb; the pulse was a little raised. Examination of the limb and wound showed no alteration. Later the pulse was again found to have increased in rate. The wound was again examined and the limb found a little swollen, a little tense, and the skin perhaps whiter than normal. After a few hours' delay the patient's condition became decidedly worse, the pulse was further accelerated, the temperature raised, and vomiting occurred. The limb was now decidedly swollen, tympanitic, and the skin showed a slight dirty yellow tint. Possibly, too, there was some crepitation. The next stage showed an aggravation of all the symptoms. The patient was decidedly ill, the pulse running, the extremities cold and blue. The limb was much swollen, the skin showed mottled blue patches and blebs, the edges of the skin about the wound were yellow. Crepitation was present at some distance from the injury. The end came suddenly, the patient perhaps losing his pain and fever, and retaining the acuteness of his mental faculties to the end.

The speaker next discussed the different appearances seen in the group type when the skin was incised. He said that he would take an imaginative case in which all types of muscle change were supposed to be present, though such changes could only be found in reality by examining many cases in different stages. (The description was illustrated by reference to the colored plates reprinted in *the Medical Bulletin* n° 2, opposite p. 116.)

The incision showed the skin and fat normal. Perhaps there was a little edema on the surface of the deep fascia. If the knife were drawn over the deep fascia, a hollow sound was produced. On incising deeper, the areolar tissue beneath was found to be edematous, and some bubbles of gas were visible. The areolar tissue between the muscles was sometimes normal filled with a yellow edema. Bubbles of gas were often present and not unusually this distention with gas took place along the great vessels. The gas found its way through the deep fascia along the perforating arteries and thus reached the subcutaneous tissues.

As to the muscles, some were normal and contractile, some dead and motionless, some dead and brick-red in color, and sometimes in these brick-red muscles bubbles of gas were seen that could be displaced by the finger up and down the muscle. These gaseous muscles were crepitant and floated in water. At a later stage these red muscles became a yellow green in color and very friable in consistency. The appearance of the green tint corresponded to the commencement of the proteolytic process. Finally the yellow green tint gave way to a black color, the change being due to the action of sulphuretted hydrogen on the iron of the hemoglobin. These last color changes were not now frequently seen as the surgeon interposed before this stage was reached. Death, too, usually took place before the " red death " stage was passed.

It was to be remembered that the changes in the muscles were usually much further advanced than those in the skin and that normal looking skin often overlay a muscle condition that was incompatible with life.

Sometimes, if the disease was traced in a wounded muscle, a yellow transverse line was seen, which was hard to the touch and which marked the advancing edge of the disease. The hardness was due to a coagulative necrosis. On one side, this line bordered on normal muscle; and toward the wound, all the color changes described above could be seen. At other times the infection spread up and down one side of a muscle and left the other side normal. The disease affected first of all the wounded muscles, spread up and down it, but found difficulty in passing from one muscle to another in a transverse direction.

The observation that gas gangrene was mostly a muscle disease was brought to the speaker's attention by Captain Kenneth Taylor, and all that the speaker had seen confirmed the observation.

In the *segmental type*, a portion of a limb died as a result of the cutting off of its blood supply and infection followed death in the same way that a coli dissemination takes place on the body after or at the moment of dissolution. The changes are the same as in the group type but the clinical phenomena of death are the first noticed.

*Treatment.* In the *group* type the wounds are to be fully opened up by adequate longitudinal incisions. Attention is focussed on the muscles. All muscles that are non-contractile or altered in color are removed, or partially resected as the condition demands. Some need to be followed to their attachments. If the condition of the patient is bad, amputation may be required as a life saving measure. The level is to be decided by the state of the greater number of the muscles. Short flaps, or circular amputations with a short skin cuff are to be used as a rule. Single muscles, if affected, can be dissected out. In cases of fracture it is usually wise to amputate at the seat of fracture.

In the *segmental* type, the level of amputation should again be determined by the state of the muscles. This can be ascertained by lateral incisions which are used to fashion the flaps when healthy muscle is reached. For gangrene of the leg, amputation through the head of the tibia is often sufficient, thus leaving an intact thigh through which a later permanent amputation can be performed.

M. E. Sacquépée, Médecin Principal de 2e Classe, then presented a paper which was read in translation by Major Blake. It was entitled " Gas Gangrene in War Wounds ".

The writer declared gaseous gangrene to be essentially a toxemia, stating that the signs of general intoxication are out of all proportion to the local lesions. These signs are a weak and rapid pulse, toxic dyspnea, earthy or icteric color of the skin, prostration, and vomiting.

In order to study the actual lesion of typical gas gangrene, we must seek it in wounds with only a small destruction of tissues. Where it occurs as a result of serious anatomical lesions, it is impossible to determine the part played by the typical gas infection.

There are 3 types of this primary gangrene : lesions of the tissues, gaseous infiltrations, and edema. They are to be found in varying proportions in all cases. The tissue lesions are chiefly muscular (hyperemia, anemia, swelling, softening, but never liquefaction). Gas infiltration occurs in small bubbles. The edema consists of clear fluid. Locally there is a peculiar odor.

*Pathogenic Study.* — Gas gangrene is known to be due to anaerobes. The chief are : B. perfringens (or B. welchii), Vibrion septique[1], and B. bellonensis. These are to be found singly or in combination in almost all wounds. Their discovery, however, is difficult, and requires a special technique.

These three species act differently in producing the disease in the guinea-pig. B. perfringens causes liquefaction of the muscle and subcutaneous tissues, producing a considerable putrid discharge in which there are pockets of gas. In some respects these lesions are like those seen in man, but in others they are different.

Vibrion septique produces muscular lesions, colored but clear edema, and gas infiltration in small bubbles, similar to the phenomena observed in men in a majority of cases.

1. S. notes that in France, since the work of Maurice Nicolle, F. Césari, et Mlle Raphael, there is a disposition to consider the B. chauvaci of symptomatic charbon to be of the same species as V. septique.

B. bellonensis acts differently, producing in the guinea-pig and rabbit, inoculated with cultures containing toxins, a colorless edema, slight gaseous infiltration, but few changes in the muscles. These lesions correspond to the edematous form of the disease (white erysipelas) as seen in man.

Clinical gas gangrene appears in several forms : generally there is a mixture of tissue lesions, of gas infiltration, and of edema. In what may be termed *diffuse gangrene*, more rarely observed, the gas infiltration assumes large proportions and becomes the chief symptom. *Septicemic gangrene*, which often supplements diffuse gangrene, is marked by a considerable edema, with comparatively little gas infiltration or muscle change. *Edematous gangrene* is a form in which general intoxication, characteristic of all three types, reaches its maximum.

The B. bellonensis is the only one of the bacteria mentioned which, if washed and injected into a rabbit, will produce the peculiar odor noticed in clinical types of the disease.

*Toxic Properties.* — The toxic property of B. perfringens has proved *in vitro* to be very weak. Vibrion septique and B. bellonensis, on the other hand, have given a very active toxin.

*Conclusions as to Sero-therapy.* — Since toxemia is the most characteristic of the clinical phenomena, it seems logical, first of all, to combat this intoxication. Vibrion septique and B. bellonensis appear to be the most active agents in producing toxemia, and, therefore, antitoxins against these organisms have been prepared. That against Vibrion septique is prepared at the Institute Pasteur by M. Jouan; that against B. bellonensis is prepared at the Laboratory of the IVth Army.

For prophylactic purposes, when only small destruction of tissues is concerned, and immediate surgical intervention is possible, a simple injection, administered as soon as possible, and repeated after 8 or 10 days, may suffice.

When, however, there is a large destruction of tissue, or a serious interruption of the blood supply, the antitoxin can be counted upon for only a slight effect. The necrosed tissues in which the organisms are active are beyond the reach of the circulation, and therefore, the serum can merely neutralize toxins as they are absorbed, and, perhaps, increase the resistance of the body. Large doses, frequently repeated, as well as local injections, both in the depths of the wound and about the margin of the dead tissues, are the only means of checking the advance of the disease under such conditions.

*Curative Treatment.* — There is little assurance that sero-therapy will do more than prevent accidents. It cannot be relied upon to check infection already started. The antitoxins for both organisms should be injected together (as also in preventive treatment), unless examination proves definitely the absence of either.

At present the following procedure has been adopted :

As soon as the diagnosis is established, large doses of both sera (40 to 60 cc. of each) are injected. These are repeated after 4 to 6 hours, and again after 16 to 24 hours.

In the presence of the edematous form, the inoculations consist of 3/4 of anti-B. bellonensis serum, and 1/4 anti-Vibrion septique serum.

The actual showing in clinical use is so far very limited but encouraging : 45 slight injuries and 21 more extensive ones have been treated prophylactically. Gangrene occurred in only 3. As a curative measure the treatment has been applied in 44 cases with marked signs of toxemia. There were 9 deaths, 2 of them due to late streptococcic infection.

Captain Henry, R. A. M. C., then spoke on the bacteriological aspects of gas gangrene. He stated that the organism now generally associated by investigators with the disease, is an anaerobe which the French call B. perfringens, and the British and Americans, B. Welchii or aerogenes capsulatus.

Since, however, it is exceedingly difficult to obtain any anaerobe in a pure culture, much of the laboratory experimentation with the organisms concerned has perhaps been erroneous or misleading. Hence the differences found in the literature upon the subject. By way of illustration, the speaker mentioned what was thought several years ago to be the discovery of a new anaerobic organism. Subsequently it was found not to be a single organism at all, but two distinct varieties in symbiosis.

The bacteriologist is at present much concerned with the question : how far do anaerobes influence each other in the wound? Do they, in symbiosis, produce results which differ from those produced when the organisms act separately?

It has been noticed that the two organisms most commonly associated in gas gangrene, namely B. welchii and B. sporogenes, seem to produce a much more serious effect when associated than when separate. B. sporogenes alone when inoculated in laboratory animals gives practically no results, and B. welchii alone produces comparatively unimportant lesions. When, however, they are injected together, the results from each are enormously increased. One might figuratively speak of B. welchii as the infantry, and B. sporogenes as the artillery, in the progress of the disease.

Much more information, however, is needed, before any satisfactory conclusions can be drawn from bacteriological investigations on the subject of anaerobes. At present the technique of bacteriological diagnosis for anaerobes is so complex that the bacteriologist can be of very little assistance to the surgeon, especially in a disease the development of which may be exceedingly rapid. Perhaps an advance in this direction might be made by the use of agglutinating sera for diagnosis.

*Serum Treatment.* — Sera have been prepared, and it is known that the enemy is employing on the western front a serum against B. welchii, Vibrion septique, and B. sporogenes. MM. Sacquépée and Weinberg have prepared similar sera. Much more experimentation, however, is still necessary before certain and efficatious results may be expected from serum treatment.

Major McNee, R. A. M. C., then spoke on the microscopic histology of gas gangrene.

Gas gangrene is most frequently associated with the long muscles, such as those of the thigh and leg. Fundamental to an understanding of the spread of the disease are the facts : (1) That the activity of the organisms starts and progresses only in dead muscle (2) that muscle fibers run longitudinally from one tendinous insertion to another. Thus very rarely is a whole segment of a thigh diseased, but instead, only a single muscle is at first likely to be invaded. Since the organism can get a start only in dead muscle, some condition must be brought about whereby a muscle is killed before the bacilli may thrive in the muscle.

It is therefore of the utmost importance that we should know what condition precedes the advance of the disease in the muscles, thus causing the necessary degeneration of the muscle by which the organism thrives and produces gas.

The speaker then considered in detail the so called " margin of advance ", illustrating his remarks by reference to colored reproductions of microscopic slides of muscle sections at the margin of advance. (These plates are reprinted in *the Medical Bulletin*, N° 2, for December, opposite page 117. They are also to be found in the *Brit. Med. Jour.* for June 2, 1917.)

At the margin of advance the organism is found always between the muscle fibers and never in them. If we follow a muscle from the seat of the injury, we notice first a gangrenous center where there is an accumulation of dead tissues and gas production, then we find a length of muscle in the condition known as " red death ", and finally we come upon the margin of advance. Here the muscle is somewhat paler and firmer than normal. If we pass beyond the margin of advance into the live muscle, we find it normal. Here the organisms may sometimes be found at a considerable distance from the margin of advance, but apparently harmless, and always between muscle fibers, never in them. A section of the healthy muscle just beyond the margin of advance will present the appearance noted in plate VIII (of the illustrations mentioned above). If a section is taken at the very edge of the margin of advance it will be noticed that some of the fibers are separated off from the interstitial connective tissue (fig. IX) by a considerable space. A section taken farther behind the margin of advance shows all of the fibers to be so separated (fig. X). As yet, however, no organisms appear to have invaded the muscle fibers, but these fibers are evidently being subjected to a necrosing process.

Just what this process may be is the vital point in the problem. What is going on in the abnormal spaces between the fibers to produce necrosis and consequently a favorable condition for the saprophytic growth of the organism. Examination of frozen muscle sections fixed by boiling and freezing, has led the speaker to believe that these spaces contain not gas, as might be supposed, but a fluid, probably of toxic proteids, produced by the organism as it feeds upon the muscle already decomposed behind the margin of advance. This fluid forces its way through the spaces between the muscle fibers and the interstitial tissues, thus bringing about the death of the fibers, which are rapidly decomposed as seen in fig. XIII, and become a favorable medium for the rapid development of the organism. At this proteolytic stage the bacilli freely invade the muscle substance, and the condition of established gangrene is produced.

In introducing CAPTAIN TAYLOR, U. S. R., who opened the *discussion*, Major Blake briefly referred to the case of hemothorax in his hospital which led Captain Taylor to observe that B. aerogenes capsulatus required necrosed muscle as the essential condition for gas production, for only when bits of dead muscle were introduced in the hemothorax fluid did the organisms in the fluid produce gas.

T. stated that among the common anaerobes found in wounds, the B. aerogenes capsulatus was much the most rapid grower and fermenter of dextrose and glycogen. As these were the chief muscle sugars, this point was important. Therefore, if gas production within muscle is an etiological factor in initiating and extending the muscle destruction, the blame must largely fall upon the Welch bacillus.

The speaker agreed with Captain Henry as to the difficulty of obtaining pure cultures of anaerobes and as to the value of cross agglutination for identifying the species.

In discussing antitoxic sera, he expressed the opinion that confidence in the efficiency of such sera depended somewhat on the interpretation of the pathology. The infection began in tissues beyond the reach of antitoxins in the blood stream, and gas distension might progressively enlarge this zone of antitoxin-free tissues by interference with the circulation. The chief value of antitoxin administration was in neutralizing such toxin as was absorbed, and so, helping to keep up the body's resistance. Nevertheless, the question of the possibility of the prophylaxis and treatment of gas gangrene by sera was very important and should be carefully investigated.

The speaker differed with M. Sacquépée's opinion that gas gangrene was essentially a toxemia. Toxemia was usually a late and secondary symptom. Unlike tetanus, gas gangrene was primarily a local disease while tetanus infection produced little local, but a profound systemic reaction. The use of antitoxins was not therefore analogous in the two diseases.

He believed the method of spread of gas gangrene in the living muscle as described by Major McNee was also an important pathological problem. Its rapidity was difficult to explain by the diffusion of a toxin, unless that toxin was driven by pressure from behind. Whatever the role played by the toxic substance, this might be formed from dead muscle by anaerobes other than the Welch bacillus — especially those of the proteolytic group. Possibly the muscle changes described by Major Mc Nee could be explained by gas diffusion alone.

The speaker believed that in the fresh wound too much attention was directed to the bacteria as the cause of the wound condition rather than to the wound condition as determining the nature of the bacterial growth. Treatment should be directed, first, to the condition of the wound (removal of the soil for the bacteria) and, secondarily, to attempts to kill the bacteria themselves. The initial infection was essentially saprophytic and did harm only when the products of bacterial growth, either toxins or gas, were retained. Then conditions might become favorable for true parasites to invade the damaged tissue.

M. Weinberg, of the Pasteur Institute, who continued the discussion, referred to the fact that his first experience with gas gangrene had been at Dr. Blake's hospital, and that as a result of this work he began his experiments in the production of a horse serum.

He stated that there is no longer any doubt that B. perfringens plays an important role in the etiology of gas gangrene. This is especially the American and German contribution to the study of the disease. The French have been inclined to attribute to Vibrion septique an important rôle. It now appears that in about 25 o/o of cases, there is confusion as to the causing agent : in only about 4 o/o of cases it is certain that V. septique is an important agent.

Additional organisms (B. œdematiens, B. bellonensis) have also been isolated from clinical types of the disease by Weinberg and Séguin, and by Sacquépée.

In about 30 o/o of cases, the speaker believes that death is due to the activity of B. œdematiens, which is very toxic.

He has prepared sera against B. perfringens, Vibrion septique, and B. œdematiens, and he considers the most efficient method of treatment to be the injection of a mixture of these three antitoxins.

In any case, there can be little doubt that the bacteriology of gas gangrene is highly important. The speaker's study of bacterial activity has led him to suppose that B. œdematiens, by reason of its highly toxic effect, produces an ideal medium for the growth and activity of B. perfringens, the chief producer of gas, and also, perhaps, for Vibrion septique. It is therefore highly important to provide an antitoxin not only against B. perfringens but also against B. œdematiens and Vibrion septique.

At the second meeting of the session, Dec. 12, Trench Fever was discussed, MAJOR MCNEE, R. A. M. C., opening the subject.

Major McNee dwelt briefly upon P. U. O. (pyrexia of undetermined origin) noting that many of the fevers thus classified at the beginning of the war have now been shown to be distinct diseases : such are those of the enteric group. In this connection he noted that the practice of preventive inoculation had made it more difficult to diagnose cases of paratyphoid as well as those of spirochaetal jaundice, formerly known as Weil's disease, the organism of which was found in 1916. Diagnosis is especially difficult in this disease since it is often unaccompanied with signs of jaundice.

At least three fourths of all cases that were originally of the P. U. O. group, are now safely classed as trench fever. The rest are still undetermined.

Trench fever proper, was first described in the British press in September 1915, by Major Graham in the *Lancet.* Graham, McNee, and Renshaw, however, had begun their observations of the new type of recurring fever considerably earlier in the spring of that year. It was soon distinguished from the enteric group by negative blood cultures and negative blood counts.

It is highly important to note, as an aid in determining the method of transmission, — a topic to be considered later, — that at first only two classes of men were affected — the men in the trenches and the men in the trench zone, that is, within a mile or two of the trenches. Only one other class was affected, the members of the Royal Army Medical Corps. So it appears that at first it affected only the men in the trenches or those coming in contact with them. Hence the men themselves came to call it "trench fever".

Naturally, with the movement of troops, it has now spread to a considerable distance back from the lines.

It seemed to affect officers and men in precisely the same proportions, and it made no difference whether they were old soldiers or new recruits.

A further point of interest is that the disease was apparently carried to Salonica by two divisions of men transferred from the western front. Major Hurst, who watched the progress of the disease in Salonica, reported its recurrence in several cases already observed by McNee in France. It was also reported as occurring soon among the enemy's troops, and was accurately described by writers in German and Austrian periodicals, especially by Werner and His.

What is known as the "short type" of the disease was first observed in France. A man was suddenly taken ill, often in the trenches; he complained of headache, and dizziness, and would sometimes fall down where he stood. The onset might be gradual, but it was often sudden. Shivery sensations were also a symptom, but these were rarely excessive. Pain was felt usually in the head, especially behind the eyes, and this was aggravated by moving from side to side. In rare cases the head pain extending into the neck and back, was so marked, that a lumbar puncture was made. Pain was also felt along the shins, and was so troublesome as to cause extreme restlessness, as patients could not keep their legs still for any length of time.

The pyrexia ranges to 103° and 104° F. : occasionally higher. In the short type, first observed, the fever continued for about 6 or 8 days, and then fell rapidly to normal, and, at the same time, the symptoms of pain, etc., disappeared.

The speaker then exhibited and explained a number of temperature charts for the short type of the sickness, noting especially that between the third and fifth days there was a marked remission of the temperature, practically to normal. During these remissions, however, the symptoms of pain, etc., continued unabated.

In most cases, convalescence was complicated by a single relapse. Such relapses usually recurred from 2 to 4 days afterwards or even longer.

In the summer of 1915 a few cases of a different or "long" type were observed, and in the winter and spring of 1915-1916 this became the prevalent form of the disease. In this, the relapsing characteristic was much more marked. In the short type, there was rarely any relapse beyond the first, but in the newer or long type, there were repeated relapses, and with each relapse the symptoms might be as strongly marked as in the first attack. During the first few days it is difficult to determine the form of the disease. In the long type, however, the initial term of the pyrexia is likely to be somewhat shorter, and there is no fall in temperature about the third day.

Suddenness is very characteristic of the relapses in the long type, and there is every appearance of a general relapsing fever. It appears impossible to establish any periodicity for the relapses, the intervals varying from 4 to 10 days. There may be as many as 9 relapses. The man frequently seems perfectly well between the bouts.

Some of the fever charts show a less regular variety of the long type, characterized by a lower temperature average and a more gradual onset. Experience and experimental work have clearly shown that all these types are merely different forms of the same disease.

Diagnosis has now become fairly easy. Malaria and dengue can be excluded. Trench fever can be distinguished from influenza by the total absence of respiratory catarrh. The diseases of the enteric group, however, are now less easily distinguished than at the beginning of the war, because now the men are inoculated for the paratyphoid as well as for the typhoid groups.

*Etiology.* — Pathological and bacteriological work have so far led to no results. Thousands of films and blood cultures have been examined in vain. Experimentation in the transmission of the disease, however, has helped to make progress towards the solution of the problem. In the summer of 1915 the speaker was allowed by the British authorities to carry out experiments of this nature.

Blood (5 cc.) taken from a patient with the short type of the fever, and then injected into the veins of a volunteer who offered himself for the experiment, caused in this man a typical case of a longer type. Similarly when blood was transfused from the second to a third man who volunteered, another case of the long type was developed. Besides proving that the disease could be transmitted by the blood, these experiments are also valuable as proof that the long and short types are merely varieties of the same disease.

Further experiments of a similar nature were carried out for other points. It was found that serum and plasma used for such experiments gave negative results, although the people inoculated were observed for months. Intramuscular injections of the blood, however, were found to produce the typical disease as well as intravenous injections.

If the corpuscles were separated and washed five times with normal saline solution and then injected, the disease was also produced.

From the whole set of experiments we may, therefore, conclude that the virus is to be found in the whole blood and is especially connected with the corpuscular elements but not with the serum or plasma. A further experiment carried out by breaking up blood corpuscles by mixing them with sand, and then extracting them with a salt solution, filtering through a Berkefeldt v filter and injecting, gave negative results. Thus the attempt to prove that the virus was a filterable one failed. All experiments on animals failed completely.

*Clinical Observation.* — The most important contribution on this topic the speaker said he could make, was based upon his study, in the spring of 1916, of 40 cases which occurred in something like an epidemic among 200 men in a field ambulance. 15 of the cases were among the personnel of a clearing station. Of the personnel, some who contracted the disease were working in light wards where there were cases of trench fever. Some men, however, worked in these wards for ten months without contracting the disease. Men engaged in such work as bearing stretchers, carrying blankets, and operating the Thresh disinfector, were also attacked. A noteworthy fact was that in 1916 the long type seemed to predominate, as has always been the case at Salonica. Also there were many cases in which relapses occurred after very long periods of complete recovery. In some cases the relapses continued for as long as a year and a half. The speaker had not observed many cases in which he believed the return of the fever was due to a new infection. One man, who developed a second attack nine months after the first, admitted that occasionally in the interval he had slight attacks of headache and pains in the legs.

*Transmission.* — At present it is of vital importance to determine the means of transmission of trench fever, and much more work remains to be done along this line. Many parasites have been suspected, but the louse has fallen under the gravest suspicion. No clear experiments have so far been made, but a great deal of cumulative evidence confirms the suspicion. Several experiments have been performed by volunteers who have allowed lice to bite them, but these have not been clean experiments. One man contracted in this way what appeared to be a case with a low temperature and no very definite relapse, but he collected the vermin from several cases and did not know what he was getting. Such experiments must be carried out by breeding lice, watching them bite a genuine case, and then watching them actually transmit it.

Various points already mentioned point, nevertheless, to the louse as the offender. In the first place, only the men in the trenches or the near neighborhood were affected. Then it was observed that officers and men were taken with the disease in about equal proportions, and it is also true that they are infested with lice to about the same extent. Furthermore, men who, like stretcher bearers, blanket carriers, and disinfectors, come in contact with the patients' clothing, often get the disease, whereas men who are in the wards, but who do not come in contact with the patients at all, are safe.

Evidence as to being bitten by lice before contracting the disease is very unreliable by way of proof. Men who come in from the trenches have usually been bitten so much that they no longer pay any attention to the fact. The hospital orderlies, however, notice their own bites. Seven of the fifteen who contracted the disease knew that they had been so bitten within a few weeks. It happened that the epidemic occurred exactly 3 weeks after the battle of Loos when the usual precautions of cleanliness were practically impossible. Thus the case against the louse is further strengthened.

Hurst at Salonica reports cases of men who seemed to be carriers of the disease. Although they completely recovered from it themselves they appear in some way to be able to communicate it to others.

It is highly important that more conclusive experimentation should be undertaken at once to prove whether the louse is responsible for the spread of this disease. It is perhaps possible to get rid of the louse, even if we cannot hope to discover the virus for this particular fever.

The length of the *incubation period* appears from the charts for the experimental cases to be from 6 to 22 days. Presuming that lice are the responsible agents, clinical observations have led Hunt and McNee to estimate the period as from 12 to 24 days. Similarly Hurst concludes from observations in Salonica that it is from 16 to 25 days. In the experiments by transmission, injections by the intravenous route produced the disease in 6 days; by the intramuscular route, in 22 days. On this point also the evidence as yet is far from conclusive.

The speaker then referred briefly to the work so far done in a wholly unsuccessful attempt to discover the virus of the disease, mentioning particularly Houston's efforts, the spirochaete theory, Pappenheimer's observations, Renaux's "*corps en demi-lune*" and Dimond's hemogregarine, which last Capt. Henry has shown to come from distilled water, and, therefore, to be in no way connected with the disease itself.

The speaker then told of his own efforts, which, he stated, have so far been equally unsuccessful. He developed a technique by which he could readily obtain cultures of the spirochaetae of Weil's disease, but this technique applied to trench fever has given no results whatever. Films have been examined in vain.

*Treatment.* — Hunt working with McNee is largely responsible for work done in this line. No specific treatment has yet proved of any avail. Salvarsan compounds and serum of convalescents have been tried in vain. Treatment is therefore entirely symptomatic. Quinine will pull down the temperature, but it appears to have no effect upon the course of the disease. Aspirin likewise does little good, and on the other hand tends to depress the patients.

In conclusion the speaker dwelt on the vast importance of the disease from the military point of view, because its ravages are among the most serious resulting from sickness. The most common diseases are : scabies, first; then I. C. T. (boils, carbuncles, and other skin infections) and next, P. U. O. These constitute about 90 o/o of all the sickness in one of the British armies. Besides being a common affection, trench fever is serious because of its disabling effects. It is by no means of short clinical duration, often lasting from 4 to 6 weeks; and, besides, it relapses for periods of many months, and, in the end, the men have to be sent home. Neurasthenia and cardiac conditions result in a good many cases. What is worse, it does not affect the weaklings of the army any more than the fine strong types.

The disease seems unlike any reported in other wars. At the time it broke out, there were many colonials among the troops, and it may have been introduced in this manner.

Major Strong, U. S. R., was called upon to open the *discussion* He said it was perhaps unnecessary for him to remark that he had been very much interested in Captain McNee's discussion of this subject. We owed to him much of our present knowledge of trench fever and particularly the demonstration that it was a transmissible infection. Until his investigations were published in February, 1916, our ideas of trench fever were very indefinite and a number of observers would not admit that it representcd a distinct, infectious disease. Major McNee had given such a complete description of trench fever on this occasion that the speaker did not feel he could add anything of importance to it and indeed had nothing new to add beyond what had been published.

He might say a word, however, about its relation to P. U. O., pyrexia of unknown or undetermined origin. A very large number of cases of fever went on the British Army records with such a diagnosis, due often to lack of opportunity to study the cases carefully, owing to the short time they are necessarily under observation and to limited laboratory facilities in the field. Cases of influenza, paratyphoid fever and, as Major McNee had said, of trench fever were often included under this term. It seemed possible also that cases of modified typhoid fever might be sometimes included. We knew that cases of typhoid fever were not altogether rare in some of the allied armies; though the figures had been available to some of us for some time, they had not been published. However, in the *Lancet*, of November 10, 1917, the following statistics of enteric fever in the French Armies were given and these might be of interest to the Society.

From November 1, 1914 to November 1, 1915, there were 87,618 cases with 8,614 deaths — a mortality of 10 o/o. It might be remembered that vaccination became compulsory in the French Army on November 28, 1914.

The speaker remembered that when he was in France early in 1915, he was able to obtain statistics of 60,000 cases of typhoid fever which had already occurred in the Army. From November 1, 1915 to November 1, 1916, there were 17,650 cases, with 700 deaths. From November 1, 1916 to September 1, 1917, there had been 2048 cases with 129 deaths. The amount, had become greatly reduced each year, probably largely through inoculation.

The number of cases of enteric fevers in the British Army had been very much less. The speaker referred only to the figures that had been recently published. In the *Lancet* for December, 1, 1917, Lieutenant Colonel Webb Johnson gave statistics of 2,500 cases admitted to his stationary hospital during the past two years with a definite diagnosis of typpoid or paratyphoid fevers. The mortality was 3.28 per cent in typhoid, among the inoculated, and in the uninoculated, 19.19 per cent. The complications occurred in the inoculated in 7.55 per cent and in the uninoculated in 35.69 per cent.

The speaker mentioned these statistics merely to point out that cases of this disease were still not very uncommon, and also to call attention to the fact that protective inoculation had often modified the disease In those in which infection had occurred. This, of course, was what might be expected.

If series of individuals, or laboratory animals, having in the beginning varying natural susceptibility for a disease, were inoculated with the same dose of vaccine, obviously various degrees of immunity against the disease would be obtained after inoculation. The chance that one of these individuals would subsequently contract the disease if exposed to infection, and the severity of the infection if it did occur, would depend upon the size of the infecting dose and the virulence of the infecting organism, in relation to his immunity.

Consequently in repeatedly inoculated individuals who contract infection from various sources, we might expect to witness great variation in the severity of the infection and great variation in the symptoms, course of the disease, serum reactions, and other laboratory tests.

In other words, we should have at times cases of modified typhoid and para-typhoid fever in men who had been repeatedly immunized by vaccination. Such cases were very difficult to detect.

For the diagnosis of enteric fever in the inoculated individual, Dryer had developed standards of agglutination test for the three organisms which had been of very great assistance in diagnosis, but it must be borne in mind that in certain cases of enteric fever which had been modified by inoculation, it might not be possible to isolate the typhoid or paratyphoid organism and that in some cases of typhoid and paratyphoid in which the organism had been isolated, the agglutination titre of the serum had not been at any time higher than in some inoculated individual *not* suffering with the disease.

So, owing to protective inoculation, we would meet with cases of enteric fever in which the diagnosis was very difficult, both from a clinical standpoint and from the laboratory tests.

Now in the early history of trench fever, it was generally admitted that the spleen was not enlarged, but recently in quite a proportion of P. U. O. cases and in trench fever cases — the speaker believed in about one-fourth — the spleen had been found

11

definitely enlarged and palpable. He believed that Sir Wilmot Herringham and others thought that trench fever had become clinically modified during the past two years.

He called attention to these difficulties in diagnosis with the idea in mind that in the future study of trench fever we must be careful not to include and confuse obscure forms of enteric fever with trench fever, as this might lead to erroneous conclusions, particularly with reference to further experimental work.

McNee's experiments, of course, showed that trench fever was distinct from even the modified forms of enteric fever; but while some of the temperature charts were distinctive, others exhibited could very well be those from cases of modified enteric fever and, in cases in addition, in which an enlarged spleen was present, one could see that differential diagnosis might be very difficult, for we had not as yet any specific laboratory method for the diagnosis of trench fever.

One of the most important unsolved problems in relation to trench fever was the method of its transmission, and this problem we expected to try to solve in the near future and we hoped to have Major McNee's cooperation and advice in this work.

Major Swift, U. S. R., continued the discussion describing his clinical experience with the disease. 20 o/o of admissions to his hospital were definite cases of trench fever. Cases seen at the present time, however, seem to differ slightly from the forms that Major Mc Nee described.

Instead of the abrupt rise and fall of temperature as seen in the " spike " indications on the charts, about 50 o/o of the cases show a continuance of the fever for 2 or 3 days. In cases with a " spike " the relapse would often be in the form of a continuous fever for several days.

Another striking characteristic in the cases is the pain at night, together with severe sweating as a consequence of which the bedclothes are often soaked. Sometimes during the day there will be two " spikes " of fever instead of the one as indicated on Major McNee's charts. In the morning the temperature may be highest, 102 to 103; at noon, normal; and in the afternoon it may rise again to 101. This is the most peculiar feature of the disease.

As to the enlargement of the spleen, which Major McNee did not mention, the speaker said he had observed it carefully, and had concluded that it was not easily confused with the typhoid spleen : it is a hard spleen like that of malaria and not soft and mushy. In most cases, even when there is an enlargement of the spleen, it is possible to rule out typhoid, although the diagnosis from paratyphoid is not so easily made.

D. A. H. (disordered action of the heart) is another feature of the disease that interests observers more and more. It manifests itself in various ways. The patient may complain of dyspnea and he will be found to have tachycardia, which may last for a few hours and then clear up. This attack may be repeated in a few days. Cardiac disorders with an extra systole are also observed.

Tachycardia that follows the disease is a third type of disorder most frequently observed. It sometimes persists for many months, although it does not necessarily follow the long type of the disease.

On the question of transmission, the speaker has noticed that his orderlies contract the disease when they have been exposed only to patients. 35 o/o of the unit have contracted the disease. It was learned that of the orderlies who denied that they had been bitten by lice, only 1 o/o developed trench fever. On the other hand, the careful experiments carried on by Dr. Pappenheimer to prove the transmission of the disease by lice have so far not been conclusive. Volunteer subjects were isolated and watched carefully for several weeks at a farmhouse before they were inoculated. Lice were allowed to feed on a patient with the "single spike" type of the disease and then on the experimental subject. The subjects were observed for four weeks or more, but up to the present time no definite results have been obtained, although interesting data have been procured.

As to the difficulty of diagnosis, the speaker noted that many cases are at first diagnosed as appendicitis which later turn out to be trench fever. There is at present a tendency among the consultants to class as trench fever cases which cannot be definitely diagnosed, and which the British call P. U. O. The large number of unwounded men who are sick with this disease is proof of its military importance. The speaker believed that the American doctors who were privileged to hear Major McNee's address would go back and watch their patients much more carefully because of Major McNee's work the results of which he had so clearly presented.

Major Roger Lee, U. S. R., stated that the experience at his base hospital was practically identical to that reported by Major McNee. Any one of the charts presented by him could be duplicated there.

On the question of etiology some experiments had been carried out, although not the brilliant ones on blood transmission performed by Major McNee. All blood studies, however, had given negative results. Spirochaetae had been found in the urine, but the speaker was convinced that they were to be found in the same proportions among normal men in the U. S. forces as among men with P. U. O.

He was under the impression that about 80 o/o of the cases vaguely "basketed" as P.U.O. were well defined cases of trench fever. None of them were fatal, but they were of widely variable duration. The first case occurred in the personnel of the hospital about 15 days after the men landed in France and 11 days after they went on active duty, at which time, probably, they were first exposed. With regard to the louse problem the speaker said: "This case, like some of the other cases which developed trench fever, was fairly characteristic. The man said that he had never been bitten by a louse. He was a college graduate, prided himself upon taking baths every day; and said that he had never been bitten by a louse, did not have a bite upon him: but upon careful scrutiny, we were actually able to demonstrate a louse upon this particular individual." Evidence as to louse bites, the speaker thought, was therefore of no importance at all.

Persistence of the fever was also noticeable, in some cases for as much as 6 months. In practically all cases the enlargement of the spleen could be proved upon careful examination. Often the symptoms were directed towards the appendix, but more frequently towards the spleen. This is likely to be tender, and, as it is also important to note, it may be enlarged either upwards or downwards.

In all particulars the speaker's experience of the last seven months corresponded with the description of the disease made by Major McNee. If the cause of the disease cannot be exactly determined, at least every effort may be made to interrupt the communication of each patient with other men. In the study of this problem, surely, lies the most important work for this association to undertake.

Major Blake then called for a general discussion, and said he would like to ask Major McNee if the disease was prevalent in other armies.

Major Lambert stated that in Italy only one case was reported at Schio, on the edge of the mountains down towards the Piave River.

Surgeon-General Thompson, B. M. S. First Army, who was requested to speak, said that he did so with feelings of great pride in the men from his Army, Colonel Wallace and Major McNee, who had introduced the two subject set forth for discussion in such masterly fashion. He was sorry he could not also claim Captain Henry.

In reverting for a moment to the subject discussed at the first meeting, Gas Gangrene, the administrative side of tke treatment of which disease it was his duty to control, he stated that three points were chiefly essential :

(1) Speedy evacuation of the wounded to the C. C. S.

(2) Ample operating accommodation there.

(3) Sufficient Surgical Teams to deal with the cases.

In this connection the advice given by Colonel Wallace (the Consulting Surgeon to the First Army) had been invaluable; and, since early operation had been proved to be essential, each new Casualty Clearing Station had been provided with from 12 to 16 operating tables, working as twins, each two accompanying one team.

The speaker agreed with Major McNee that " Inflammation of Connective Tissue " (I. C. T.) was one of the most serious causes of wastage to the Army at present.

He spoke especially of the dangers of overtreatment with sulphur, drugs, etc., and stated that one skin specialist suggested the rubbing of men with strong sulphur twice daily for four days without giving a bath between the applications, but the speaker did not allow this method to be put into practice in his corps.

Major Blake said he would like to know if anything was being done to get rid of lice. He thought others might like to ask questions. Major McNee was then asked to classify the type of fever without the shin pains. Major Lambert said that he had seen in Belgium a number of cases of unjaundiced Weil's disease, and asked Major McNee how it could be differentiated from trench fever. Another member asked if there was any characteristic blood picture of trench fever to help in differentiating it from typhoid; also whether cases with all the symptoms of trench fever, but with practically no fever, should be considered as trench fever.

Major McNee then proceeded to answer the questions proposed. As to the enlargement of the spleen, he said that during the first year of the disease Sir Wilmot Herringham and he had not found it to be enlarged, although it was a routine point in all the examinations they made. Later, however, while working with Captain Hunt, he had found cases with the spleen enlarged, hard, and palpable. In other cases, apparently identical, no enlargement of the spleen could be detected. This might be due to the difference in the topography of the body. In men with a wide costal arch it was easy to detect, but in men with a narrow chest, it was often difficult. It is certainly clear that in many cases the spleen is enlarged, and palpable. At autopsies, however, during the last three years it has been observed that various organs in soldiers are found to be greatly enlarged. The spleen has been found enlarged in many soldiers who have died suddenly from wounds. The thyroid is astonishingly enlarged, sometimes to 100 grams, although its normal weight is from 18 to 20 grams. It is frequently found to weigh from 30 to 40 grams.

There is a type of fever, about which the speaker said he was not yet clear in his own mind. It was not typhoid and not trench fever. He did not wish to say any more about it at present.

He said that there was, as Major Strong intimated, a great deal of confusion at present resulting from inoculation against the paratyphoid group in addition to the usual typhoid inoculation. He thought, however, that the actual amount of P. U. O. belonging to the enteric group was small.

The incidence of the disease in other armies has been fairly general. It occurred at about the same time as in our own. It has not been recognized to a very large extent in the Belgian and French armies. It was not very common in the Russian armies; neither was nephritis. The Austrian and German armies have any amount of this disease on all their fronts.

Probably the cases of fever without shin pains and of shin pains without the fever, belong to trench fever. It is far from a definite and constant type of disease. Sometimes the pains have occurred in the buttocks and the backs of the legs. Why it should be generally noticed in the shins the speaker could not say.

The differentiation of this disease from the unjaundiced form of Weil's disease was often impossible. If, however, there are other cases of unjaundiced Weil's disease in the same place, there may be a presumption against the diagnosis of trench fever. Possibly it might be proved by trying to produce Weil's disease in the guinea-pig. Many such cases have been tested, however, without any effect.

In the blood picture there is nothing characteristic, except, perhaps, a tendency towards mono-leucocytosis. In the " spike " type there is a definite leucocytosis. Anemia was common at the beginning of the disease, due, perhaps, to a poor supply of vegetables.

The attempt to rid the armies of lice is purely administrative.

It is admittedly a difficult task, for the trenches of both sides are louse-ridden from end to end. Washing and change of clothing seems to afford small relief. It has been noted by Sir Wilmot Herringham, however, that in a division which he investigated, the regiments that were especially cleanly, had much less P. U. O. than the others.

Major Blake announced that the executive committee of the Association had already appointed a special committee to undertake the study of trench fever in cooperation with our British friends. Trench fever and nephritis would be so studied.

ANNOUNCEMENT. — The Research Committee wishes to announce that it will be glad to receive reprints of articles on war medicine, surgery, and hygiene for the reference library at 6, rue Piccini, Paris. They may be directed to the secretary of the Committee at this address. Extra copies will be loaned in response to requests received for literature upon special topics.

